# THE ROLE OF CULTURE AND LANGUAGE IN DEPRESSION CARE EXPERIENCES AMONG OLDER LATINAS/OS AND HEALTH CARE PROVIDERS

**DOI:** 10.1093/geroni/igz038.2171

**Published:** 2019-11-08

**Authors:** Maria P Aranda, Janelle Christensen, Iris Aguilar

**Affiliations:** 1 University of Southern California, Los Angeles, California, United States; 2 Florida SouthWestern State College, Fort Meyers, Florida, United States; 3 USC Edward R. Roybal Institute on Aging, Los Angeles, California, United States

## Abstract

Older Latinas/os face significant difficulties in accessing quality depression treatment due to cultural and language barriers. In our study, Programa Esperanza (a randomized behavioral trial), we conducted after-treatment, in-depth interviews with two groups: 21 low-income Latinas/os (55+) receiving psychosocial depression care (Problem Solving Treatment, and Psychoeducation) in a health care setting, and 22 staff (interventionists, supervisors) participating in study implementation. Speaking the same language was highlighted by both groups as an overarching factor in effective depression care, although matching providers and patients by country of origin was considered less a priority than a shared language. The mechanisms by which language serves as a facilitator of effective care were highlighted: fomenting rapport; facilitating the expression of feelings; shortening initial relationship-building; understanding nuanced words and linguistic expressions; etc. Similarly, we found that shared culture included themes around intervention uptake, decreased stigma, increased advocacy, enhanced identification of coping strategies, etc. Unlike patients, providers were more likely to speak in diverse narratives of “them,” and “us.” Given that depression is still a stigmatizing disorder in our society, asking for help and receiving quality care remain significant challenges for older persons in general, and older underrepresented minorities, in particular. Our work signals the importance of differentiating language from culture in intervention development for older, primarily Spanish-speaking Latinas/os with high medical comorbidity.

